# Combined associations of regular exercise and work-related moderate-to-vigorous physical activity with occupational stress responses: a cross-sectional study

**DOI:** 10.3389/fspor.2024.1386775

**Published:** 2024-05-09

**Authors:** Takafumi Abe, Kenta Okuyama, Atsushi Motohiro, Daijo Shiratsuchi, Minoru Isomura

**Affiliations:** ^1^ Center for Community-Based Healthcare Research and Education (CoHRE), Head Office for Research and Academic Information, Shimane University, Shimane, Japan; ^2^ Center for Primary Health Care Research, Department of Clinical Sciences Malmö, Lund University, Malmö, Sweden; ^3^ Canvas Inc., Shimane, Japan; ^4^ Department of Physical Therapy, School of Health Sciences, Faculty of Medicine, Kagoshima University, Kagoshima, Japan; ^5^ Faculty of Human Sciences, Shimane University, Shimane, Japan

**Keywords:** brief job stress questionnaire, stress check program, physical activity, mental health, paradox

## Abstract

**Objective:**

The association between work-related moderate-to-vigorous physical activity (MVPA) and higher levels of stress response is recognized, but whether this association is moderated by regular exercise remains unclear. This cross-sectional study investigated whether exercise-based physical activity (PA) associates with lower levels of stress responses moderated by work-related MVPA.

**Methods:**

The study participants comprised 863 workers from 35 small and medium-sized enterprises in Shimane prefecture, Japan, collected through convenient sampling from April 2021 to August 2022. The Brief Job Stress Questionnaire was used to assess stress responses. Work-related MVPA and exercise-based PA were measured using questionnaires. Multiple linear regression was used to analyze the combined variables of work-related MVPA and exercise-based PA. The reference group had no weekly exercise-based PA and >60 min of work-related MVPA.

**Results:**

When work-related MVPA exceeded 60 min/day, flexibility activity or walking for ≥5 days/week (*B *= −3.53, 95% CI = −5.96, −1.11; *B *= −2.53, 95% CI = −4.90, −0.16) and muscle-strengthening activity 1–3 times/week (*B *= −3.52, 95% CI = −6.91, −0.12) were significantly associated with lower psychological stress response. Flexibility activity (*B *= −1.74, 95% CI = −3.01, −0.46) showed a similar link with physical stress response. When work-related MVPA was below 60 min/day, flexibility activity (*B *= −3.23, 95% CI = −6.01, −0.44; *B *= −3.29, 95% CI = −5.94, −0.63) or walking (*B *= −4.03, 95% CI = −6.62, −1.45; *B *= −3.10, 95% CI = −5.76, −0.44) practice 1–4 times/week and ≥5 times/week was significantly associated with lower psychological stress response.

**Conclusion:**

Exercise-based PA greatly and consistently associates with a lower level of stress responses moderated by work-related MVPA.

## 1 Introduction

In the workplace, excessive stress causes poor physical and mental health, including depression and cardiovascular diseases (1, 2), resulting in an increase in sick leaves (3). In the Japanese context, nearly 53.3% of the workers experience excessive stress (4), making it crucial to implement suitable stress management strategies to mitigate occupational stress responses.

Although White et al. reported that leisure-time physical activity (PA) was beneficial for mental health (5), high work-related PA was associated with poor mental health. Holtermann et al. further discussed the health paradox of work-related PA (6). In summary, people who engage in high levels of leisure-time PA are less likely to experience long-term sick leaves, whereas those with high levels of work-related PA face an elevated risk of long-term sick leaves.

In a previous study of ours, while a higher level of work-related moderate-to-vigorous PA (MVPA) increased the odds of occupational stress responses measured by the Brief Job Stress questionnaire, regular exercise reduced such odds (7). However, the extent to which regular exercise is beneficial for the stress responses moderated by work-related MVPA remains unclear. Given this background, this cross-sectional study investigated what type of exercise-based PA—flexibility, muscle strengthening, or walking—is associated with lower levels of stress responses moderated by work-related MVPA.

## 2 Materials and methods

### 2.1 Study design and participants

**Figure 1:**
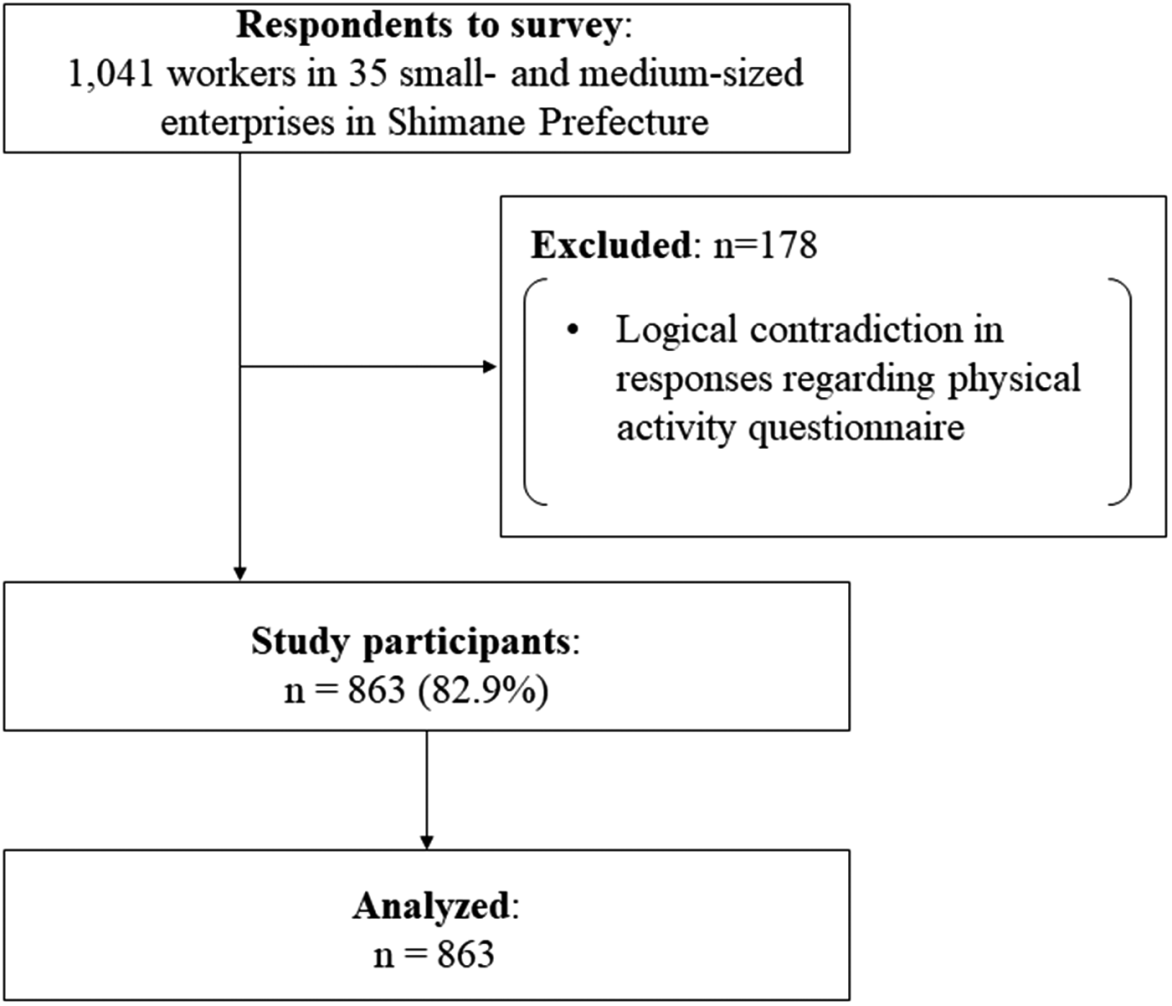
Study participant flowchart.

An overview of the study participants was reported in a previous study (7). A total of 1,041 workers from 35 small and medium enterprises in Shimane Prefecture participated through convenient sampling from April 2021 to August 2022. After excluding 178 individuals who exhibited logical contradictions in their responses to the PA questionnaire, the data of 863 participants were analyzed (Figure 1). Informed consent was obtained from all participants prior to the commencement of the study. The study protocol was approved by the Research Ethics Committee for Human Subjects of Shimane University Faculty of Human Sciences (#2022–2). All procedures were performed in accordance with the tenets of the Declaration of Helsinki.

### 2.2 Measurements

The Brief Job Stress Questionnaire (also known as BJSQ) was used to assess occupational stress, as divided into the psychological stress response (18 items) and physical stress response (11 items) subscales (8). Each item was rated on a four-point Likert scale, total scores were calculated as the sum of the scores for both subscales, and a higher total score implied a higher level of stress response (8). The scores for psychological and physical stress responses demonstrated good internal consistency (Cronbach's alpha ≥0.839), test-retest reliability (Pearson's correlation coefficient ≥0.689), and factor-based validity (first factor ≥39.4%) in a previous study (9). This study used scores as continuous variables.

The two types of PA were measured using a questionnaire. First, work-related MVPA was assessed using the Work-related Physical Activity Questionnaire (also known as WPAQ) (10). The time spent in MVPA (min/day) was calculated as the sum of the time spent walking and engaging in heavy loads during work, and MVPA was divided into two groups based on the median value (60 min/day). A previous study reported the acceptable reliability and validity of the Work-related Physical Activity Questionnaire (10). Second, flexibility, muscle-strengthening activities, and walking were used as exercise-based PA; the frequency (times/week) of the three activities were assessed using a modified questionnaire based on previous studies (11, 12). Previous studies have demonstrated that moderate flexibility and muscle-strengthening activities show acceptable test-retest reliability. Additionally, walking exhibited satisfactory test-retest reliability and criterion-related validity. While previous studies have investigated frequency (day/week) and amount of practice (minutes), this study focused solely on weekly frequency. Exercise-based PAs were categorized into three groups: no activity (0 times per week = the reference group), low activity (below the median frequency), and high activity (at or above the median frequency).

Information regarding sex, age, educational attainment, household income, employment status, managerial position, smoking habits, and alcohol consumption habits were gathered through a self-administered questionnaire. Body mass index (BMI in kg/m^2^) was computed using the participants’ self-reported weight and height measurements.

### 2.3 Statistical analysis

The characteristics of the study participants were indicated based on whether their work-related MVPA was ≤60 min/day and >60 min/day. Categorical data were presented as counts and percentages, while continuous data were represented using the median and interquartile range (IQR). Multiple linear regression analyses were performed to estimate the unstandardized regression coefficient (*B*) and 95% confidence interval (CI) of each occupational stress factor for the combined variables of work-related MVPA and exercise-based PA. To clarify whether stress responses based on the amount of work-related MVPA are alleviated by exercise-based PA, six groups were set up to determine the combined conditions of work-related MVPA (≤60 min/day and >60 min/day) across three categories of frequency in each exercise-based PA (flexibility, muscle-strengthening activities, and walking), with the group reporting >60 min/day of work-related MVPA and zero time/week of each exercise-based PA used as the reference group.

Analyses were conducted using crude and adjusted models. For the adjusted model, the analysis was adjusted for sex, age, BMI, educational attainment, household income, employment status, managerial position, smoking habits, and alcohol consumption. Missing information about independent and dependent variables, which ranged from 0.2%–18.8% (7), was processed using multiple imputations under the missing-at-random assumption. Each imputation was based on the regression models of the analyzed variables. The 20 imputed datasets were analyzed independently and combined for inference, accounting for the variability in imputation (13). All statistical analyses were performed using SPSS version 29 (IBM Corp., Armonk, NY, USA).

## 3 Results

Table 1 shows the characteristics of the study participants. This study included 37.9% of female. The median (IQR) age was 43 (32, 53) years. The median (IQR) of psychological and physical stress responses were 36 (29, 43) and 18 (15, 22) points, respectively. The work-related MVPA were 60 (27, 144) min/day. respectively. The median frequency of flexibility and muscle-strengthening activity, and walking were 0 (0, 5), 0 (0, 0), and 0 (0, 5) times/week, respectively.

**Table 1:** Characteristics of study participants (n = 863).

Variables		Total	Work-related MVP, ≤60 min/day	Work-related MVPA, >60 min/day
		*n* = 863	*n* = 444	*n* = 419
		Median (IQR) or *n* (%)	Median (IQR) or *n* (%)	Median (IQR) or *n* (%)
Sex, *n* (%)	Female	327 (37.9)	180 (40.5)	147 (35.1)
	Male	526 (61.0)	261 (58.8)	265 (63.2)
	Other	1 (0.1)	0 (0)	1 (0.2)
	Missing data	9 (1.0)	3 (0.7)	6 (1.4)
Age; years, median (IQR)		43 (32, 53)	43 (32, 52)	43 (33, 53)
	Missing data, *n* (%)	26 (3.0)	13 (2.9)	13 (3.1)
BMI; kg/m^2^, median (IQR)		22.1 (20.2, 24.5)	22.0 (20.1, 24.7)	22.0 (20.0, 24.3)
	Missing data, *n* (%)	68 (7.9)	38 (8.6)	30 (7.2)
Educational attainment, *n* (%)	High school or less	382 (44.3)	150 (33.8)	232 (55.4)
	More than high school	454 (52.6)	280 (63.1)	174 (41.5)
	Other	7 (0.8)	1 (0.2)	6 (1.4)
	Missing data	20 (2.3)	13 (2.9)	7 (1.7)
Household income, *n* (%)	Less than 6 million yen	569 (65.9)	285 (64.2)	284 (67.8)
	More than 6 million yen	173 (20.0)	99 (22.3)	74 (17.7)
	Unsure	91 (10.5)	40 (9.0)	51 (12.2)
	Missing data	30 (3.5)	20 (4.5)	10 (2.4)
Employment status, *n* (%)	Full-time	658 (76.2)	334 (75.2)	324 (77.3)
	Other	188 (21.8)	98 (22.1)	90 (21.5)
	Missing data	17 (2.0)	12 (2.7)	5 (1.2)
Managerial position, *n* (%)	Yes	288 (33.4)	156 (35.1)	132 (31.5)
	No	538 (62.3)	269 (60.6)	269 (64.2)
	Missing data	37 (4.3)	19 (4.3)	18 (4.3)
Smoking status, *n* (%)	Yes	167 (19.4)	72 (16.2)	95 (22.7)
	No	693 (80.3)	370 (83.3)	323 (77.1)
	Missing data	3 (0.3)	2 (0.5)	1 (0.2)
Alcohol consumption, *n* (%)	Every day or sometimes	465 (53.9)	240 (54.1)	225 (53.7)
	Hardly or do not drink	396 (45.9)	203 (45.7)	193 (46.1)
	Missing data	2 (0.2)	1 (0.2)	1 (0.2)
Stress responses				
Psychological stress response, points, median (IQR)		36 (29, 43)	35 (29, 43)	37 (29, 43)
	Missing data, *n* (%)	162 (18.8)	81 (18.2)	81 (19.3)
Physical stress response, points, median (IQR)		18 (15, 22)	18 (15, 23)	18 (15, 22)
	Missing data, *n* (%)	15 (1.7)	6 (1.4)	9 (2.1)
Work-related MVPA; minutes/day, median (IQR)		60 (27, 144)	27 (12, 48)	144 (96, 216)
Exercise-based physical activity				
Flexibility activity; times/week, median (IQR)		0 (0, 5)	0 (0, 4)	0 (0, 5)
	Missing data, *n* (%)	3 (0.3)	2 (0.5)	1 (0.2)
Muscle-strengthening activity; times/week, median (IQR)		0 (0, 0)	0 (0, 0)	0 (0, 0)
Walking; times/week, median (IQR)		0 (0, 5)	0 (0, 3)	1 (0, 5)
	Missing data, *n* (%)	2 (0.2)	2 (0.5)	0 (0)

IQR, interquartile range; BMI, body mass index; MVPA, moderate to vigorous physical activity.

Table 2 shows the associations among work-related MVPA, exercise-based PA, and occupational stress responses. In the adjusted model and regarding psychological stress responses, considering work-related MVPA >60 min/day and flexibility activity practiced ≥5 times/week, there was a significant association with lower psychological stress responses (*B *= −3.53, 95% CI = −5.96, −1.11). Similarly, for work-related MVPA of ≤60 min/day and flexibility activities 1–4 times/week (*B *= −3.23, 95% CI = −6.01, −0.44) and ≥5 times/week (*B *= −3.29, 95% CI = −5.94, −0.63), there were significant associations with lower psychological stress responses. For work-related MVPA >60 min/day and muscle-strengthening activity practice 1–3 times/week, there was a significant association with lower psychological stress responses (*B *= −3.52, 95% CI = −6.91, −0.12). For work-related MVPA >60 min/day and walking practice ≥5 times/week, there was a significant association with lower psychological stress responses (*B *= −2.53, 95% CI = −4.90, −0.16). Regarding work-related MVPA ≤60 min/day and walking practice 1–4 times/week (*B *= −4.03, 95% CI = −6.62, −1.45) and ≥5 times/week (*B *= −3.10, 95% CI = −5.76, −0.44), there were significant associations with lower psychological stress responses.

**Table 2:** Association between exercise-based and work-related moderate-to-vigorous physical activity with occupational stress response among workers.

		Psychological stress response						Physical stress response					
		Crude model			Adjusted model			Crude model			Adjusted model		
		*B*	(95% CI)	*P*-value	*B*	(95% CI)	*P*-value	*B*	(95% CI)	*P*-value	*B*	(95% CI)	*P*-value
Work-related MVPA	Flexibility activity												
>60 min/day	Group 1 (0 time/week)	Reference			Reference			Reference			Reference		
	Group 2 (1–4 times/week)	−1.82	(−4.58, 0.93)	0.19	−1.93	(−4.63, 0.77)	0.16	−0.51	(−1.96, 0.95)	0.50	−0.40	(−1.86, 1.06)	0.59
	Group 3 (More than 5 times/week)	−3.89	(−6.37, −1.42)	<0.01	−3.53	(−5.96, −1.11)	<0.01	−1.87	(−3.13, −0.61)	<0.01	−1.74	(−3.01, −0.46)	<0.01
≤60 min/day	Group 4 (0 time/week)	−2.40	(−4.38, −0.42)	0.02	−1.90	(−3.89, 0.09)	0.06	−0.70	(−1.73, 0.34)	0.19	−0.62	(−1.68, 0.45)	0.26
	Group 5 (1–4 times/week)	−3.67	(−6.47, −0.87)	0.01	−3.23	(−6.01, −0.44)	0.02	−1.20	(−2.63, 0.24)	0.10	−1.22	(−2.69, 0.24)	0.10
	Group 6 (More than 5 times/week)	−3.84	(−6.53, −1.16)	<0.01	−3.29	(−5.94, −0.63)	0.02	−1.34	(−2.67, −0.01)	0.05	−1.22	(−2.57, 0.13)	0.08
Work-related MVPA	Muscle-strengthening activity												
>60 min/day	Group 1 (0 time/week)	Reference			Reference			Reference			Reference		
	Group 2 (1–3 times/week)	−2.86	(−6.29, 0.57)	0.10	−3.52	(−6.91, −0.12)	0.04	−1.65	(−3.44, 0.13)	0.07	−1.72	(−3.52, 0.08)	0.06
	Group 3 (More than 4 times/week)	0.61	(−2.77, 3.99)	0.72	0.41	(−2.93, 3.75)	0.81	−0.74	(−2.46, 0.99)	0.40	−0.78	(−2.51, 0.96)	0.38
≤60 min/day	Group 4 (0 time/week)	−1.50	(−3.16, 0.16)	0.08	−1.07	(−2.77, 0.62)	0.22	−0.43	(−1.28, 0.43)	0.33	−0.40	(−1.28, 0.48)	0.37
	Group 5 (1–3 times/week)	−3.17	(−6.88, 0.55)	0.10	−3.65	(−7.32, 0.01)	0.05	−0.65	(−2.50, 1.20)	0.49	−0.86	(−2.75, 1.03)	0.37
	Group 6 (More than 4 times/week)	−1.87	(−5.56, 1.82)	0.32	−1.73	(−5.39, 1.93)	0.35	−1.41	(−3.22, 0.39)	0.13	−1.42	(−3.25, 0.41)	0.13
Work-related MVPA	Walking												
>60 min/day	Group 1 (0 time/week)	Reference			Reference			Reference			Reference		
	Group 2 (1–4 times/week)	−0.83	(−3.75, 2.09)	0.58	−0.85	(−3.77, 2.07)	0.57	−0.42	(−1.94, 1.10)	0.59	−0.25	(−1.78, 1.28)	0.75
	Group 3 (More than 5 times/week)	−2.70	(−5.08, −0.32)	0.03	−2.53	(−4.90, −0.16)	0.04	−0.87	(−2.09, 0.36)	0.17	−0.69	(−1.92, 0.54)	0.27
≤60 min/day	Group 4 (0 time/week)	−1.02	(−3.05, 1.01)	0.33	−0.67	(−2.74, 1.39)	0.52	−0.09	(−1.15, 0.98)	0.88	−0.01	(−1.10, 1.09)	0.99
	Group 5 (1–4 times/week)	−4.65	(−7.23, −2.08)	<0.01	−4.03	(−6.62, −1.45)	<0.01	−1.39	(−2.71, −0.06)	0.04	−1.25	(−2.60, 0.10)	0.07
	Group 6 (More than 5 times/week)	−3.61	(−6.28, −0.94)	<0.01	−3.10	(−5.76, −0.44)	0.02	−1.15	(−2.50, 0.20)	0.09	−1.05	(−2.42, 0.33)	0.14

Each physical activity indicator was examined separately using multiple linear regression. For the adjusted model, the analysis was adjusted for sex, age, body mass index, educational attainment, household income, employment status, managerial position, smoking habits, and alcohol-drinking habits.

MVPA, moderate-to-vigorous physical activity; B, unstandardized regression coefficients; CI, confidence interval.

For physical stress responses, there was a significant association when considering work-related MVPA >60 min/day and flexibility activity ≥5 times/week (*B *= −1.74, 95% CI = −3.01, −0.46). For work-related MVPA of ≤60 min/day and flexibility activity, there was no association with physical stress responses. No associations were found between work-related MVPA and muscle-strengthening activities or walking and physical stress responses.

## 4 Discussion

On examining the combined association between work-and exercise-based PA and occupational stress responses among workers, our findings suggest that regular exercise may reduce stress responses, regardless of work-related MVPA. When the daily duration of work-related MVPA exceeded 60 min and the engagement in flexible or aerobic activities was five or more times per week, there was an association with lower psychological stress responses. However, in cases where work-related MVPA was low, even a weekly frequency of once or more of flexible and aerobic activities was associated with lower psychological stress responses. Additionally, a beneficial association with psychological stress responses was observed when engaging in muscle-strengthening activities one to three times per week, specifically in situations with high levels of work-related MVPA. Regarding physical stress responses, flexible activity conducted five or more times per week may be beneficial only when occupational MVPA is high. When work-related MVPA levels are low, engaging in frequent flexibility and aerobic activities once or more may benefit stress responses. However, when work-related MVPA is high, there is a potential association with elevated stress responses, suggesting that flexibility and aerobic activities should occur at least five times a week.

Our study revealed that engaging in flexibility activity correlates with lower psychological and physical stress responses. While the American College of Sports Medicine (ACSM) recommends flexibility exercises twice a week (14), our research suggests that engaging in flexibility activities more than five times a week with high levels of work-related MVPA, significantly reduces psychological and physical stress responses. Hence, it may be worthwhile to consider a higher frequency than the recommended minimum. Although different from our study design, Corey et al. found that psychosocial stress improved with restorative yoga (control group) compared to a low-impact stretching at least 3 times per week for at least 30 min per session intervention (stretching group) in individuals with metabolic syndrome (15). After a year of monitoring, the stretching group showed notable reductions in levels of cortisol in saliva, severity of chronic stress, and stress perceptions, when compared to the control group. Chronic stress-induced cortisol dysfunction may contribute to the onset of persistent pain (16). Although it is widely recognized that flexibility exercises are advantageous for preventing and alleviating musculoskeletal pain, a prior review also highlighted their potential usefulness in managing work-related musculoskeletal pain (17). Flexibility activities may improve both psychological and physical stress responses.

When doing muscle-strengthening activities one to three times per week, an association with lower psychological stress responses was found, particularly in situations involving high levels of work-related MVPA. However, no association between muscle-strengthening activity four or more times per week and psychological stress response. Kamada et al. reported a J-shaped relationship between the duration of muscle-strengthening activity and mortality, with the lowest mortality rate observed at 82 min per week (18). While our study aligned closely with the World Health Organization's recommended frequency of muscle-strengthening activity (at least twice a week) (19), further investigation is warranted regarding the association between the frequency and volume of muscle-strengthening activity and health outcomes, including stress responses. We hypothesized how muscle-strengthening activity may mitigate psychological stress response in cases of high work-related MVPA. One potential mechanism could be the improvement of muscle imbalances and reduction of work-related burdens (20). Additionally, muscle-strengthening activity can promote the secretion of serotonin, stabilizing mood, and potentially aiding sleep induction. As an interventional study, Becker et al. investigated the stress responses induced by resistance training (21), showing that salivary cortisol levels decreased during upper body strength training sessions, after which an improvement in positive effect was observed.

Aerobic exercise, particularly walking, has long been recognized for its psychological and physical health advantages. The ACSM recommends performing aerobic exercises three to five times a week (14). Amid real-life stressors, the group that underwent aerobic exercise training exhibited noticeably diminished physiological stress reactivity in the autonomic nervous system, specifically in terms of heart rate variability, when compared to the control group (22). von Haaren et al.'s interventional study showed that the aerobic exercise training group significantly reduced physiological stress reactivity of the autonomic nervous system (heart rate variability) compared to the control group (22). Furthermore, aerobic exercise may promote the secretion of serotonin as a neural regulatory mechanism (23), potentially contributing to the alleviation of stress and fatigue.

### 4.1 Limitations

This study has several limitations. First, the use of a cross-sectional design prevents us from making causal inferences regarding the relationship between PA and occupational stress. Second, the study participants were not randomly selected, potentially introducing selection bias. Third, the frequencies of work- and exercise-based physical activities were categorized differently, with daily and weekly distinctions, and a comprehensive assessment of all PA levels was not possible. Finally, we were unable to account for the effects of unmeasured variables such as work environment (e.g., job type and industry), work-related factors (e.g., workload and work pace), and contextual factors (e.g., bullying, harassment, and violence). Based on this, further intervention studies are necessary to elucidate whether various types of exercise-based PA during work-related MVPA are effective in reducing both psychological and physical stress and to explore the underlying mechanisms.

## 5 Conclusion

In conclusion, our findings indicate that exercise-based PA is greatly and consistently associated with lower levels of stress responses moderated by work-related MVPA. Further intervention studies are required to establish causal relationships.

## Data Availability

The raw data supporting the conclusions of this article will be made available by the authors upon reasonable request. Requests to access the datasets should be directed to the corresponding author.
